# Unraveling the Roles of Vascular Proteins Using Proteomics

**DOI:** 10.3390/molecules26030667

**Published:** 2021-01-27

**Authors:** Yan Liu, Tianbao Lin, Maria Valderrama Valencia, Cankui Zhang, Zhiqiang Lv

**Affiliations:** 1 Institute of Sericulture and Tea, Zhejiang Academy of Agricultural Sciences, Hangzhou 310021, China; mayanly@sina.com (Y.L.); kissumbra@126.com (T.L.); 2 Departamento Académico de Biología–Universidad Nacional de San Agustin de Arequipa Nro117, Arequipa 04000, Peru; mvalderramav@unsa.edu.pe; 3 Department of Agronomy and Purdue Center for Plant Biology, Purdue University, West Lafayette, IN 47907, USA

**Keywords:** xylem proteins, phloem proteins, sieve elements, vasculature

## Abstract

Vascular bundles play important roles in transporting nutrients, growth signals, amino acids, and proteins between aerial and underground tissues. In order to understand these sophisticated processes, a comprehensive analysis of the roles of the components located in the vascular tissues is required. A great deal of data has been obtained from proteomic analyses of vascular tissues in plants, which mainly aim to identify the proteins moving through the vascular tissues. Here, different aspects of the phloem and xylem proteins are reviewed, including their collection methods, and their main biological roles in growth, and biotic and abiotic stress responses. The study of vascular proteomics shows great potential to contribute to our understanding of the biological mechanisms related to development and defense in plants.

## 1. Introduction

Higher plants contain vascular bundles that connect all of their organs and act as a long-distance communication system to transport carbohydrates, nutrients, and growth signals throughout the plant, in order to regulate growth and development [1]. Plant vascular bundles contain two major types of transport units, the xylem and the phloem, with the apoplast compartment acting as an interface between them. Xylem tissues are composed of tracheary elements, parenchyma cells, and fiber cells that provide physical support for plant growth in woody plants [2,3]. During differentiation, tracheary elements lose their nuclei and cellular contents, which leaves a hollow tube that becomes part of the xylem vessel, which is used to transport the water and minerals taken up by the roots from the soil. In contrast, the phloem tissue facilitates the movement of photosynthates and other macromolecules among different organs within the plant. Phloem tissues include phloem parenchyma cells (PPCs), companion cells (CCs), and sieve elements (SEs). During differentiation, the SE undergoes selective autophagy, which results in the breakdown of the nucleus, tonoplast, and some other organelles such as ribosomes, Golgi, and microtubules. Consequently, the enucleate SE loses its protein biosynthesis ability and has limited metabolic activity, and it depends on companion cells for many of its functions via the establishment of a functional SE–CC complex between the two cell types [2,4].

In recent years, evidence has accumulated that various components, including hormones, mRNAs, amino acids, proteins, and lipidic molecules, might participate in the coordination of the developmental and physiological events at the whole-plant level [5,6]. Many highly-informative data sets derived from microarrays and the recent sequencing-based omics analyses have been obtained from plants. Xylem and phloem tissues have been shown to harbor various RNAs and proteins [7]. Specific information related to the vascular RNA repertoire is available in recent reviews [8,9]. In addition, thousands of vascular proteins have been identified from plants, including cucurbits, rice, *Ricinus*, and *Brassica napus* and *Populus*, using 1-D PAGE [10,11,12], 2-D PAGE [5,13,14,15], iTRAQ [16], and three-dimensional gel electrophoresis [17]. In this review, we focus on the vasculature located proteins with functions in growth, development, defense, and biotic and abiotic stresses. We also discuss the technical advances and challenges in the area of vascular sampling.

## 2. Sample Collection Methods from Vascular Tissues

### 2.1. Xylem Samples

Xylem sap has frequently been obtained by decapitating plant shoots and collecting from the cut shoot due to root pressure [18]. This can be performed for most monocots and many dicots, including *Solanum lycopersicum*, *Brassica napus* and *B. oleracea*, because their stems are large enough to collect sufficient volumes of xylem sap [2,10,13]. The cutting of plant stems followed by the application of external pressure using a Scholander chamber may lead to the excessive release of intracellular proteins. A prerequisite for the use of this technique is that the studied plant needs to have turgid stems. Another weakness related to this technique is the underestimation of the concentrations of the components in the xylem sap due to the dilution effect of the pressure.

The collection of exudates facilitated by root pressure is more suitable for non-woody plants. For woody plants, e.g., *Populus* and *Vitis*, additional manipulations, such as external bark removal, is required [18,19]. When a piece of bark is removed from a tree, new periderm is formed and wound cambium is developed from the callus on the surface of the secondary xylem. New phloem is subsequently formed from the wound cambium [20]. After the separation of the phloem and cambium from the stem, the wood-forming tissue can be collected from the exposed surface by scraping it with a razor blade. This scraped secondary xylem is expected to contain cell types that include secondary cell-wall–forming xylem vessels and fibers. Changes in protein expression patterns corresponding to the different stages of secondary vascular system regeneration provide the opportunity to monitor the biological events during the different stages of wood formation [21,22].

### 2.2. Phloem Samples

Phloem sap is much more difficult to obtain than xylem sap in most plant species, and the major challenge of phloem sap proteomics is therefore to obtain sufficient authentic materials for analysis. Several different methods have been used, and their feasibilities largely depend on the plant species of interest.

#### 2.2.1. Stylectomy

Some plants exude sap spontaneously when their phloem tissues are cut. Phloem sap can be collected by making shallow incisions into the bark of the hypocotyl. Each cut severs one-quarter to one-third of the bark, and attention should be paid to avoid damaging the underlying woody tissues (xylem). This stylectomy method has been used in rape [23], cucumber [24], melon [25], mulberry [26], apple trees [27], and tomato [28].

Recently, an alternative method of sap collection has been reported, which involves peeling off the outermost (phloem) layer, and placing the peels into a plastic centrifuge tube with stainless steel balls, followed by centrifugation to acquire the phloem sap [28]. This method is straightforward and rapid, but it is not feasible with most plants because the accumulation of callose and P-proteins at the wound site can block the flow of sap.

#### 2.2.2. Insect Stylectomy

Another classical method for phloem sampling is to take advantage of insects, such as aphids and leaf-hoppers, that feed on phloem. In order to collect the phloem sap using this method, the stylet is first severed from the insect body [29]. Due to the high turgor pressure of the SEs, the stylet that is left behind exudes phloem sap for a period of time. The sap can be collected and used for transcriptomic and proteomic analysis. An improved technique has been developed recently using aphid stylectomy with many plants. In this technique, aphids are placed in sealed cages, which are flooded with water-saturated silicon oil immediately after the micro-cauterization of the stylets. The phloem sap exuded from the stylet is then collected with a capillary connected to a pump [30]. In addition, phloem sap has also been obtained by puncturing the inflorescence stems of plants, such as those of *Brassicaceae* species, with a small hypodermic needle [23], and concentrating the phloem sample using centrifugation with a molecular weight cutoff (MWCO) of 10,000 Daltons (Da) [31].

The insect stylectomy method provides relatively-pure phloem sap. However, it remains challenging due to its labor-intensive nature, the low amount of sap collected, and its limitation to certain insect–plant combinations. The collected sap might be contaminated with a trivial amount of xylem components, because many phloem sap-feeders also feed on xylem sap occasionally [27].

#### 2.2.3. EDTA-Facilitated Exudation

EDTA-facilitated exudation is the most popular method; it enables phloem sap to be collected from many plants, including those species that do not naturally exude sap [5,32,33]. In this method, phloem sap is collected by submerging the ends of severed petioles in an EDTA-containing solution for several hours. The EDTA inhibits the accumulation of callose and P-proteins in the phloem sieve tube, and results in a continuous outflow of the SE contents into the EDTA solution. The disadvantage of this method is that samples are easily contaminated with the contents of xylem and other non-phloem tissues, which probably come from the damaging of plant tissues by the EDTA [34].

### 2.3. Laser Capture Microdissection (LCM)

A different strategy from the aforementioned phloem sap analysis methods is to directly collect the vascular tissues before the proteomics profiling [35]. Vascular bundles from *Arabidopsis thaliana* have been collected by laser micro-dissection/pressure catapulting (LMPC) and subjected to high-efficiency liquid chromatography (LC) in conjunction with tandem mass spectrometry (LC/MS) [36]. A similar strategy that dissected the vascular components from the stem of broccoli was also reported [37]. A more extensive adoption of LCM for species that are less feasible for the sap methods will lead to promising discoveries related to vascular-tissue–specific proteomics.

## 3. Xylem Proteins

Peroxidase was the first protein identified from apple xylem sap. Since then, the proteins present in xylem sap have been isolated in various plant species (Table 1), and many of them are metabolic, glycolytic enzymes, and cell wall repair and defense proteins [18,38,39]. In *Brassica napus*, although the size of the proteins ranged from 1.5 kDa to 228.4 kDa, the majority (90%) of them had molecular masses between 5 and 50 kDa [40]. Many of the xylem-sap proteins contained predicted *N*-terminal signals, and derive from two different sources. One derives from autolyzed protoplasts that are released into the xylem, and the other contains proteins that are synthesized and secreted from stele cells in the root [38,41,42].

Out of the 258 peptides identified from the xylem sap of cotton seedlings, 90.31% were secreted proteins [43]. However, in woody species, such as poplar, most xylem-sap proteins were not identified as being secreted based on an informatic analysis, because only 33 proteins (34.0%) were predicted to possess an *N*-terminal signal peptide that directed them for secretion [18]. The anatomical differences between herbaceous and woody species may be one of the factors leading to this distinction. For example, a high percentage of proteins was also identified as being secreted in *Brassica napus*, another herbaceous plant [15], whereas a separate profiling of the proteins in *Populus*, a woody plant, demonstrated a similar tendency to the early one [18,44]. Two intriguing proteins, *β*-1,3-glucanase and peroxidase, with known functions in plant defense were identified from the xylem in response to *Xylella fastidiosa* in two different studies [19,45]. Another factor that contributed to the large differences between various studies was related to the diverse experimental procedures that were used. For example, the identification of nearly 4283 proteins with a shotgun method from *Populus* was probably due to the successful enrichment of the nuclei from the xylem tissue [44], but it is also legitimate to identify 77 proteins—a much smaller number—with a low throughput 2-D technique in the same species [18]. The comparison of the 4283 and 77 proteins showed that 57 proteins appeared in both studies, indicating the existence of some conserved xylem proteins across the different experiments. Similar to our analysis and these two studies, a previous report showed that the functional domains of genes in the xylem transcriptome are moderately-to-highly conserved in vascular plants [46].

**Table 1:** Overview of the xylem proteomics.

Plant Species	Treatment Condition	The Collection of Material	The Collection of Technique	Separation Method	Number of Identified Proteins	Reference
**Normal growth condition**						
Rice		Xylem sap	stem de-topped	1DE-LC, 2D-LC, HPLC-Chip-MS	118 *	[47]
Maize		Xylem sap	stem de-topped	2-DE and nano ESI-MS/MS	154	[14]
*Brassica oleracea*		Xylem sap	stem de-topped	SDS-PAGE and LC-MS/MS	189	[41]
*Brassica napus*		Xylem sap	stem de-topped	2-DE and ESI-Q-TOF tandem MS	69	[15]
*Glycine max*		Xylem sap	stem de-topped	2-DE, MALDI-TOF MS and LC–MS/MS	38	[48]
Cotton		Xylem sap	stem de-topped	shotgun HPLC-ESI-MS/MS	455	[10]
*Populus*		Xylem sap	stem de-topped and bark stripped	2-DE and LC-MS/MS	77	[18]
*Populus*		Developing xylem	bark peeled and developing tissue scraped	shotgun LC-MS/MS	4283	[44]
*Populus*		Xylem and phloem tissues	upper sides of bent stem	shotgun LC-MS/MS	3510	[11]
transgenic *Populus*		Xylem tissues	stem pieces	ultraperformance LC/ quadrupole time-of-flight MS	1486	[21]
**Abiotic stress condition**						
Maize	Drought	Xylem sap	stem de-topped	2-DE and LC-MS/MS	39 *	[49]
Maize	N under- or over-supply	Xylem sap	stem de-topped	2-DE and MALDI-TOF/TOF	230 (23 *)	[13]
Cotton	potassium-deficiency	Xylem sap	stem de-topped	SDS-PAGE and UPLC-MS/MS	258	[43]
*Brassica napus*	cadmium stress	Xylem sap	stem de-topped	shotgun LC-MS/MS	672 (73 *)	[40]
Tomato	Fe and Mn deficiencies	Xylem sap	stem de-topped	shotgun LC-MS/MS	643 (119 * Fe deficiency, 118 * Mn deficiency)	[42]
**Biotic stress condition**						
Tomato	*Fusarium oxysporum*	Xylem sap	stem de-topped	SDS-PAGE and MS/MS	**	[50]
Tomato	*Fusarium oxysporum*	Xylem sap	stem de-topped	2-DE, MALDI-TOF MS and LC QTOF MS/MS	33 *	[51]
*Brassica oleracea*	*Fusarium oxysporum f.sp. conlutinans (Foc)*	Xylem sap	stem de-topped	shotgun LC-MS/MS	About 200	[38]
Grape	*Xylella fastidiosa*	Xylem sap	Stem cutting and phloem peeled	2-DE and LC-MS/MS	10 (3 *)	[19]
Grape	*Xylella fastidiosa*	Xylem tissue	stem pieces peeled off phloem	2-DE and MALDI/TOF MS	>200 (17 *)	[45]
Rice	*Xanthomonas oryzae pv. oryzae*	Xylem sap	cutting leaves	SDS-PAGE and MALDI/TOF MS	324 (64 *)	[52]
*Glycine max*	*Bradyrhizobium japonicum strain CB 1809*	Xylem sap	Hypocotyl and epicotyl decapitated	1-DE, 2-DE, LC-MS/MS and MALDI-TOF/TOF	24	[53]

* Different proteins; ** no statistical data.

### 3.1. Cell-Wall Metabolism and Development

Among the 244 differentially-displayed proteins in poplar, 27 of them—including glycoside hydrolases and polygalacturonases—were found to be involved in secondary wall formation [22]. The main function of these glycine-rich proteins is to degrade primary cell walls, a process that is essential for the development of tracheary elements during cell death [18,49]. Several other candidate proteins related to the regulation of secondary cell walls or wood formation were also identified from xylem tissues in poplar, including SND1, NST1, CtCP and CHB3-SWI/SNF [44]. PtMC13, PtMC14 and AtMC9 homologs in hybrid aspen (*Populus tremula × tremuloides*) were involved in the proteolytic processes and cell death of xylem elements [21].

### 3.2. Biotic and Abiotic Stress

The composition of xylem sap changes in response to the environment. The components distributed in the sap may play important roles in intra- and inter-cellular communication. It is known that various xylem sap proteins contribute to plant defense responses.

#### 3.2.1. Abiotic Stress

The xylem proteome contains various proteins that are involved in redox homeostasis and plant defense, and proteases such as peroxidase and chitinase, germin-like proteins, pathogenesis-related (PR) proteins, and phytoalexin phenylpropanoid synthesis-related proteins. These molecules are believed to form a defense barrier and root-to-shoot signaling against abiotic stress.

##### Water Deficiency

The flux of the xylem in plants grown under drought and salinization differs from that in normal conditions. A total of 39 differentially-expressed proteins were identified from xylem sap in response to water stress in maize. These differentially-expressed proteins comprised two biological categories. The first category is related to cell wall metabolism, including peroxidase, xyloglucan endotransglycosylase, polygalacturonase inhibitor, and pectin methylesterase. The second category is related to plant defense mechanisms, including thaumatin-like pathogenesis-related protein, zeatin-like protein, cupin family protein, putative germin A, class IV chitinase, and *β*-1,3-glucanase [49]. It has been proven that Clavata 3/Embryo-Suppounding Region-related 25 (CLE25) protein is expressed in vascular tissues, and moves from the roots to the leaves in response to dehydration stress. CLE25, together with BARELY ANY MERISTEM (BAM) receptors, functions in the receptor-mediated signal transduction cascades to modulate diverse development and physiological processes in *Arabidopsis* [54].

##### Abnormal Nutrition Supply

Compared to normal growth conditions, low potassium (K) resulted in the accumulation of multiple environmental-stress-related proteins in xylem sap, including isoforms of peroxidases and chitinases, protease inhibitor, non-specific lipid-transfer protein, and histone H4 [43]. In total, 23 proteins were found to be differentially accumulated in the xylem under varying N supply [13]. Most of the genes encoding these proteins had higher expression levels in the roots, indicating the root origin of these root-to-shoot mobile proteins via the xylem. Iron (Fe) and Mn are cofactors for many enzymes, and they play pivotal roles in photosynthesis. Iron and Mn deficiencies were reported to lead to an alteration of 119 and 118 xylem-sap proteins, respectively. The main related molecular pathways are protein metabolism, stress/oxidoreductases, and cell wall modifications [42].

##### Heavy Metals Stress

The profiling of the proteins in xylem sap can be used to elucidate the mechanisms underlying the responses of plants to heavy-metal stress. After exposure to Cadmium (Cd) in *Brassica napus*, 73 xylem proteins were identified to be differentially accumulated, which were mainly involved in protein synthesis/degradation, stress/oxido-reductase, cell wall metabolism, and carbohydrate metabolism [40]. Reactive Oxygen Species (ROS) is overproduced in plants exposed to Cd, and the over-accumulated BnPDFL and CAP/oxido-reductase-related proteins in the xylem may enable the plant to deal with the oxidative stress more efficiently in the xylem or leaves via long-distance transmission. The OsZIP7 protein is expressed in vascular bundle parenchyma cells in roots and nodes. It plays an important role in xylem loading in roots and inter-vascular transfer in nodes. The protein preferentially transports Zn and Cd to the developing tissues and grains in rice [55]. A defensing-like protein, CAL1, is predominantly expressed in the root exodermises and xylem parenchyma cells, and is proposed to chelate Cd in the cytosol, thereby lowering the cytosolic Cd concentration and reducing the long-distance Cd transport via xylem vessels [40].

#### 3.2.2. Biotic Stress

The xylem constitutes an environment in which microorganisms can thrive. Changes in the xylem proteome during plant–microbe interactions include pathogenesis-related proteins, peroxidases, and proteins that affect the vascular system, such as the formation of tyloses, callose, and secondary cell wall deposition [56]. For example, the *β*-1,3-glucanase, 10-deacetyl baccatin III-10-*O*-acetyl transferase-like, COP9, and aspartyl protease nepenthesin precursor proteins were identified to be involved in the tolerance against Pierce′s Disease in grape [45]. A glucanase (GH17) and PR4 were identified in response to infection by *Verticillum longisporum* in the xylem sap of *B. napus* [41].

Several fungal species—e.g., *Verticillium* spp. and *Fusarium oxysporum*—that cause wilt disease can colonize the plant’s vascular system. During the process of colonization in tomato xylem vessels, the fungus *Fusarium oxysporum f.sp. lycopersici* (FOL) secretes small proteins, such as SIX (Secreted in Xylem), arabinanase, and oxidoreductase [57]. The plant response towards FOL includes a strong alteration in the accumulation of pathogenesis-related protein, PR-5x [58], and XSP10 (similar to lipid-transfer proteins (PR14)) within the xylem vessels [38]. *Verticillium* spp. is a vascular wilt pathogen that can infect many dicotyledonous plant species. VnaSSP4.2 is a 14-kDa cysteine-containing protein that was recently discovered as a *V. nonalfalfae* virulence effector protein in the xylem sap of infected hops [59]. Several ATG8-family–interacting proteins that are responsive to *V. dahliae* infection represent candidates for more detailed studies on autophagy-mediated plant immunity [56].

Pierce′s disease (PD) is a significant threat to grape production. It is caused by *Xylella fastidiosa,* a bacteria residing in the xylem. A comparison of the xylem proteins in the PD-tolerant muscadine grape and PD-susceptible bunch grape identified a number of proteins associated with energy metabolism, disease resistance, stress-related functions, biosynthesis, protein processing and degradation, signal transduction, cell-wall biogenesis, and ROS detoxification. The *β*-1,3-glucanase, 10-deacetylbaccatin III-10-*O*-acetyl transferase-like, COP9, and aspartyl protease nepenthesin precursor proteins were present in PD-tolerant muscadine grape, but were absent in PD-susceptible bunch grape [45]. The identification of the cell wall modification enzymes indicated the importance of cell wall alteration in response to the virus. In agreement with this assumption, experimental evidence has shown secondary vessel-wall thickening during Pierce’s infection in grapevines [60].

Bacterial leaf blight in rice is caused by *Xanthomonas oryzae pv. oryzae* (*Xoo*), and could lead to significant yield losses worldwide. The proteomics analysis of the xylem sap collected from the infected leaves identified 324 unique proteins, including various known or putative virulence factors [52]. Interestingly, the mutant of one of the identified proteins, protein U, exhibited a reduced mean lesion length in comparison with the wild type under infection. Future studies involving the mutant analysis of other protein candidates may shed further light on the molecular mechanism of the infection process.

## 4. Phloem Proteins

The proteins in the phloem exudate range from 10–200 kDa in size, but the majority of them are within the ranges of 10–40 kDa and 60–70 kDa [61]. This is in agreement with the size exclusion limit of plasmodesmata in the collection phloem of leaves, as proteins with larger molecular mass are more difficult to move from the companion cells to the associated sieve element [62]. To date, several hundred phloem proteins (Table 2) have been found to be relevant to metabolic processes, signaling transduction, structural formation, and stress responses in a variety of species [17,37]. The greatest number of identified phloem proteins in *B. napus* were involved in modification/turnover and general metabolism, followed by those involved in redox homeostasis and defense, and cell structural components [23]. In order to understand the similarities of the phloem proteins extracted from different species, Lopez-Cobollo et al. (2016) reported that 78% of pumpkin phloem proteins and 55% of cucumber phloem proteins [63] also appeared in the exudate analysis from pumpkin in a previous study [5]. However, only a 31% similarity was shared between the *B. napus* phloem sap proteome from the *B. oleracea* phloem tissue proteome [63]. The high similarities among the cucurbit species might be related to the similar phloem sap collection method, whereas the relatively low similarities between the two *Brassicaceae* species might be related to the different approaches used for the phloem component collection.

In one of the recent studies, both transcriptome and proteome analysis were conducted on phloem sap from the mulberry trees infected with phytoplasma. Among the total of 955 unigenes and 136 proteins that were differentially expressed, only 14 overlapped between the two profiling methods. More interestingly, 4 of the 14 common ones even had an opposite expression alteration tendency [26]. Post-translational/transcriptional mechanisms or analytical bias was proposed to be the reason. In addition, it was also postulated that mRNAs and proteins in the phloem saps might function in multiple pathways with regard to the whole plant physiology.

It has long been believed that mRNAs in the phloem sieve tube don’t degrade, but our recent heterografting study showed that degradation did take place, because 1096 mRNA were found to be transferred from the companion cell to the sieve element, but only 242 of them finally arrived in the roots [57]. It remains intriguing whether some of the aforementioned phloem proteins with modification/turnover metabolism or RNA binding play roles in regulating long-distance mRNA movement and degradation.

**Table 2:** Overview of the phloem proteomics.

Plant Species	Treatment Condition	Collecting Sources	Collecting Methods	Proteomics Method	Number of Identified Proteins	Reference
**Normal growth condition**						
Pumpkin		phloem sap	EDTA-facilitated	SDS-PAGE, Micro-LC/LC–MS/MS	47	[64]
Pumpkin		phloem sap	EDTA-facilitated	SDS-PAGE, LC-MS/MS	1121	[5]
*Brassica.napus*		phloem sap	stem punctured	2-DE and 1-DE, MALDI-MS, ESI-MS/MS	140	[23]
*Lupinus tesenis*		phloem sap	stem punctured	2-DE and 1-DE, MaLDI-MS and ESI-MS/MS	54	[65]
*Brassica napus*		phloem sap	stem punctured	3-DE and MALDI-TOF MS	>100	[17]
Curcurbit		phloem sap	stem punctured	2-DE and 1-DE, ESI-Q-TOF-MS/MS	45 *	[24]
*Brassica oleracea*		phloem SE	stem sliced	SDS and CHAPSO PAGE, LC-MS/MS	127	[37]
Pumpkin/Cucumber		Fascicular phloem	stem micro-dissected	SDS-PAGE, LC MS/MS	248/303	[66]
Rice		phloem tissues	insect laser method	1-DE + 1D-LC MS/MS, 2D-LC MS/MS	107 *	[47]
Rice		small vein	leaf anatomy	iTRAQ, nano-LC-MS/MS	1333 (294 *)	[67]
**Abiotic stress condition**						
Cucumber	NaCl stress	phloem sap	EDTA-facilitated	iTRAQ, LC-ESI-MS/MS	745 (111 *)	[16]
Tomato	drought	phloem sap	EDTA-facilitated	LC-MS/MS	2558 (169 *)	[68]
*Populus*	wounding	phloem saps	phloem flow from a cut stem into solution	2-DE, LC-MS/MS	48	[69]
*Brassica oleracea*	different cutting periods	phloem tissues	branch stripped	2-DE and MALDI/TOF- MS	40 (14 *)	[70]
**Biotic stress condition**						
*Arabidopsis*	*Pseudomonas syringae pv tomato strains*	phloem exudates	petiole cut	LC-MS/MS	62 *	[33]
Tomato Beefsteak	*verticillium dahiae incompatible*	stem extraction	stems excised, phloem peeled	LC-MS/MS	32 *	[28]
Tomato Early Pak	*verticillium dahiae compatible*	stem extraction	stems excised, phloem peeled	LC-MS/MS	30 *	[28]
Citrus	*Candidatus Liberibacter asiaticus*	bark (vascular)	outer bark centrifuged	1-DE, LC-MS/MS	692 *	[71]
Melon	*Melon necrotic spot virus*	phloem sap	stem cut	2D-DIGE, MS/MS and LC–MS/MS	19 *	[25]
Melon	*cucumber mosaic virus*	phloem sap	petiole cut	2D, MALDI-TOF MS/MS	**	[72]
Ash tree	emerald ash borer (*Agrilus planipennis*)	phloem tissues	branches stripped of leaves	DIGE and nano-LC-MS/MS	355	[73]
Rice	brown plant-hopper	phloem sap	EDTA-facilitated	iTRAQ and nano-LC ESI QqTOF MS	238	[74]

* Different proteins; ** no statistical data.

### 4.1. RNA-Binding Proteins

Studies have shown that RNA molecules are transported in the phloem in the form of complexes with RNA-binding proteins (RBPs) [75]. RBPs contain motifs, such as CU-rich polypyrimidine-binding regions, which facilitate RNA binding and transport. A set of RBPs, including CmPP16 and CmPSRP1, provided the foundation for the identification of the protein components that bind RNA and function within an RNA-based communication network [23,76]. The phloem localization of PP16 has been verified in cucurbits by both immune-localization and in situ localization. It has been suggested that PP16 plays an important role in long-distance signaling function in phloem [77]. Another cucurbit phloem serpin-1 (CmPS1), a protease inhibitor that is mobile in the phloem, is also able to bind RNA in the phloem [75]. Similar to CmPS-1, CsPS-1 in cucumber (*Cucumis sativus*) also accumulates in phloem exudates. PS1 was hypothesized to be involved in protein turnover and degradation [78]. It would be intriguing to elucidate whether this protein has a role in RNA turnover.

### 4.2. Structural and Developmental Proteins

Several structural proteins have been identified in the phloem, including lectins, cell-wall–associated proteins and cytoskeletal proteins, and proteins involved in metal homeostasis, and hormone synthesis/degradation or turnover. Flowering Locus T (FT) identified from phloem sap promotes floral induction [23]. The Altered Phloem Development (APD) transcription factor is required for phloem formation [79]. Auxin receptor protein TIR1/AFB mediates PIN repolarization, and plays roles in the regeneration of vascular strands and leaf venation [80]. Calmodulin-signal–related proteins, such as phloem protein 1 (PP1) and PP2, function in the blocking of damaged sieve tubes. An essential step for plant carbon distribution is through the phloem involved in different phloem proteins, such as transporters and ATPase. Proteins related to photosynthesis and carbon fixation are accumulated at high levels in leaves. Some of these proteins are markers for small-vein initiation in rice leaves, including sucrose degradation in newly-initiated veins, and starch synthesis in mature veins [67].

### 4.3. Stress Response Proteins

The majority of the phloem proteins that have been identified to date are involved in stress, redox regulation, and defense responses. Dehydration-related proteins—such as dehydrin, RD (responsive to dehydration) and ERD (early-responsive to dehydration) proteins—are partially involved in defense [56]. Raffinose family oligosaccharides (RFOs) participate in various aspects of plant physiology. As a key enzyme for stachyose biosynthesis, cucumber stachyose synthase (CsSTS), a protein localized in the phloem companion cells, has been shown to be involved in phloem loading, carbohydrate distribution, and tolerance to low-temperature stress in cucumber seedlings [81]. We recently revealed that a series of genes related to the biosynthesis and metabolism of RFOs were enriched in mulberry under combined salt and drought stress [82]. Whether the phloem harboring proteins related to RFO biosynthesis and metabolism are involved in the combined salinity and drought stresses is worthy of future exploration.

#### 4.3.1. Abiotic Stress

The abundance of 169 phloem proteins changed significantly in response to drought stress. These included proteins involved in lipid metabolism, chaperone-mediated protein folding, carboxylic acid metabolism, abscise acid signaling, cytokinin biosynthesis, amino-acid metabolism, cell-wall organization, and a mitogen-activated protein kinase [68]. Two of the proteins, glucose-6-phosphate dehydrogenase 6 (G6PD6) and the lactate/malate dehydrogenase family protein (At4G17260), have been previously shown to be involved in salinity-associated ROS metabolism [5,83]. In addition, a bioinformatics analysis implied that phloem proteins are responsive to salinity, with altered processes in metabolism, photosynthesis, amino-acid metabolism, translation, and chlorophyll biosynthesis.

Iron (Fe) deficiency elicited major changes in stress and redox homeostasis, C metabolism, photosynthesis, and proteolysis. Two low–molecular-weight Fe-binding proteins, a photosystem I iron-sulfur center protein and a metallothionein-like type 2B protein, were identified in phloem sap. Metallothionein is a small cysteine-rich protein with a strong binding capacity for heavy metals. It is conserved and has been identified in the phloem sap of rice, *R. communis*, and *Lupinus texensis* [65], suggesting a conserved role of this phloem protein across different species. The alteration of the iron–sulfur center protein indicated that low iron might have led to a change in the abundance of this iron-containing protein in the phloem photosystem.

#### 4.3.2. Biotic Stress

Plants have evolved a system in which infection by pests can be sensed by non-infected sites. This process involves long-distance signaling via the phloem. In addition, the organic compounds transported through the phloem can serve as nutrients, and can be exploited by a large number of pathogens, nematodes, aphids, and phloem-feeding insects.

##### Defense against Fungi

Proteins secreted by vascular wilt fungi play roles in pathogenicity and plant immunity. The growth of *Fusarium* spp. was strictly limited to the phloem in American ginseng (*Panax quinquefolius* L.) [84]. The citrus industry is currently under threat by Huanglongbing (HLB), a very devastating and widespread citrus disease. The disease is caused by *Candidatus Liberibacter asiaticus* (*CLas*), a bacterium that specifically resides in the phloem. Quantitative proteomics revealed significant changes in the citrus proteome after *CLas* inoculation. For example, defense-related proteins, such as peroxidases, proteases and protease inhibitors, were induced [71]. These proteins are promising candidates for further functional analysis into diverse responses to HLB across various environmental conditions and citrus genotypes.

##### Defense against Viruses

The phloem provides the most rapid way for proteins involved in systemic defense responses to move throughout the host. Many viruses use long-distance phloem translocation to spread throughout the whole plant. Forty-four potato proteins and one viral protein were identified to be associated with Potato leaf roll virus (PLRV) isolated from the infected phloem [85]. Two *Arabidopsis thaliana* proteins restricted TEV movement 1 (RTM1) and RTM2, which restrict the mobility of potyviruses in the phloem, conferred resistance to several potyviruses, and were specifically localized to the SEs [37]. Chaperonin-containing T-complex polypeptide 1 subunit 8 was identified to interact with the PLRV; the KNOTTED 1 transcription factor was suggested to assist in virus transport [86]. In contrast, it was reported that KNOTTED 1-like homeobox transcription factor (NTH202) interacted with Bt56 protein, suppressed whitefly-induced SA accumulation, and decreased whitefly performance [87]. The detailed mechanisms of the regulation of the plant defense associated with KNOTTED 1 transcription factor interaction warrant further investigation.

Twenty-five proteins involved in redox homeostasis and cell death were accumulated in the phloem during *Melon necrotic spot virus* (MNSV) infection in melon according to 2D gel analysis. Of these, two proteins—carboxylesterase and fumarylacetoacetate hydrolase—have been previously shown to be negative regulators of cell death [25]. The two stress-related proteins, pop3/SP1 and thaumatin-like protein (TLP), were differentially-expressed 24 h after wounding [69]. The manipulation of the expression level of these genes—e.g., the downregulation of the negative regulator of cell death in the phloem—in plants may lead to increased resistance to the virus.

Infection with cauliflower mosaic virus (CMV) induced the accumulation of a few phloem proteins, including major latex protein (MLP), anenolase, a translationally controlled tumor protein homolog (TCTP), the heat-shock cognate protein 70, and an additional unknown stress-associated protein [72]. In a separate study, Calcineurin B-like-interacting protein kinase 7 (CIPK7) was localized in or near plasmodesmata. It can interact with the read-through domain (RTD) of Turnip yellows polerovirus (TYPV), and can block the long-distance movement of the virus [86]. These studies provide evidence that phloem plays a pivotal role in the mediation of the host responses during infection.

##### Defense against Insects

Aphids are phloem-feeding insects that damage plants, and they are major agricultural pests. About 150 candidate proteins were identified from the salivary proteome of the cowpea aphid by liquid chromatography tandem mass spectrometry (LC-MS/MS), including diacetyl/l-xylulose reductase (DCXR), which is a novel plant defense protein against aphid pests [88]. In legumes, the spindle-like protein bodies (forisomes) that found in the sieve tubes are probably involved in plant defense by rapidly occluding sieve pores in response to aphid attack [89]. *Pinellia pedatisecta agglutinin* (*ppa*) encoding mannose binding lectin was reported to be highly anti-nutritional and toxic to various phloem-feeding insects [90]. Future efforts to increase the phloem-specific accumulation of these proteins with biotechnological approaches may block the feeding of, and increase the resistance to, aphids.

The emerald ash borer (*Agrilus planipennis*) is an invasive wood-boring beetle that has killed millions of ash trees in North America. A comparison of the phloem proteomes between strains of ash that were resistant and susceptible to emerald ash borer identified proteins associated with resistance, which included a PR-10 protein, an aspartic protease, a phenylcoumaran benzylic ether reductase (PCBER), and a thylakoid-bound ascorbate peroxidase [73]. A similar method revealed that the abundance of 238 proteins was significantly altered among the phloem exudates of resistant and susceptible rice plants following brown planthopper (BPH) infestation. These proteins were involved in multiple pathways, including defense signal transduction, redox regulation, and carbohydrate and protein metabolism, as well as cell structural proteins. A successive quantitative PCR and an in situ mRNA hybridization verified that most of the identified phloem proteins were indeed located in the phloem [74]. The rice hoja blanca virus (RHBV), transmitted by the planthopper insect, was also studied by comparative proteomic tools for the analysis of the molecular response mechanisms in new crop varieties [91]. Further functional characterization of some of the promising proteins may provide candidate proteins for future biotechnological manipulation.

## 5. Mobile Vascular Proteins

### 5.1. Xylem Mobile Proteins

There is increasing awareness that proteomics is an effective but challenging approach for the identification of long-distance mobile proteins mediating organ-to-organ communication in higher plants. Recent studies have revealed two soybean xylem proteins—lipid transfer protein and Kunitz trypsin inhibitor (KTI)—with known functions in plant signaling [53]. In another study, it was found that CLAVATA3/embryo-surrounding region (CLE) and C-terminally encoded peptide (CEP), two short peptides, were synthesized in the roots and moved to shoot tissues via the xylem in response to plant–microbe interactions and nitrogen starvation, respectively [92]. Whether increasing the abundance of these xylem transmitting proteins—e.g., CEP—will lead to increased shoot responses remains an interesting scientific question that is worthy of exploration.

### 5.2. Phloem Mobile Proteins

It has been proposed that some phloem-enriched proteins and compounds with viscous, sticky and toxic properties act as components of a viral surveillance/resistance mechanism, as well as integrators of processes at the whole-plant level. The presence of a high diversity of biochemically-active proteins in phloem sap suggests that they might be involved in the coordination of metabolism, development, and defense responses at the whole-plant level. Phloem proteins, such as CmPP16-1 and CmPP16-2, appear to move differentially to the root, which provides an insight into their function as component of the RNA-based systemic signaling mechanism [93]. The SlCyp1 protein is mobile in the phloem, and is transported from the shoot to the root to induce lateral root formation. Therefore, SlCyp1 integrates shoot photosynthesis with requirements for access to water and mineral nutrients in the roots [94]. Some plant pathogens, such as viruses and bacteria, can also spread systemically via the phloem throughout a host plant. The function of the phloem as the tissue for viral long-distance movement might be related to its susceptibility to infection, and the easy amplification of infection along the movement of the virus via the CC–SE complexes. The proteomic analysis of grafted plants has shown that proteins associated with pathogen infection were more highly represented in tolerant rootstocks than in sensitive ones, suggesting the direct involvement of plant defense mechanisms in response to a pathogen challenge in the phloem [95]. These findings highlight the importance of the phloem for the investigation of host–pathogen interactions, as well as plant development. In line with these findings, it has long been desirable to develop phloem-mobile fungicides that can be applied to the leaves in order to control root or vascular pathogens.

### 5.3. Factors Confering Mobility to Proteins

Hetero-grafted plant systems involve two different species, and have been used to identify the long-distance movement of phloem-mobile molecules—e.g., proteins—in response to different stresses. It has been found that some proteins in CCs are specifically targeted to the sieve tube lumen for long-distance trafficking. In addition to this, certain proteins also enter the translocation stream “by accident” [96]. A few prior studies have also proposed that some phloem-mobile proteins might be synthesized directly in the SE, which contains Golgi, endosomes, and small vacuoles. However, further studies are needed to show whether this is real, because it has been shown that the activities of ribosomal proteins are suppressed by phloem exudates. How macromolecules get into the phloem—e.g., whether this is a highly regulated process or it just occurs accidentally due to abundance or molecular mass—remains a subject of active research. A few possibilities have been raised: (1) protein mobility is probably due to certain protein domains that confer mobility and/or protein stability. The detection of FT in the phloem sap, and the successive grafting experiment showed that FT constitutes an important part of a mobile florigen. Six amino acid residues located on the FT protein were found to be essential in conferring mobility to Twin Sister of Ft (TSF). A substitution of one or more of these amino acids may cause a conformational change or the dysfunction of the protein molecules, which may further affect the interaction between the protein, the target, and its mobility [97]. (2) The mobility of a protein might be related to its molecular weight. The size exclusion limit of plasmodesmata in the phloem of leaves is reported to be under 70 kDa [62,98]. Therefore, proteins with greater molecular weights may be more difficult to transfer from companion cells to the sieve element. (3) Long-distance transport is dependent on efficient inter-organ interaction and communication. For example, the lipid-transfer protein, which is defective in induced resistance 1 (DIR1), is a member of a protein complex that can be transported to distant leaves via the phloem during systemic acquired resistance, and a deficiency of DIR1 reduces cell-to-cell movement [33].

## 6. Future Prospects

Proteomic knowledge is useful for breeding programs, and may facilitate the improvement of commercial crop production. Because vascular tissue is not easily accessible, and because sap composition varies with the temperature, time of the year, light conditions, water stress, and soil nutrient status [63], more attention should be paid to the improvement of the accuracy of methods for exudate analyses. Highly sensitive analytical methods will allow a comprehensive identification of phloem proteins in the future. For example: (1) combinatorial hexapeptide ligand libraries (CPLL; such as ProteoMiner) are a time- and cost-effective tool to reduce the dynamic range of protein concentrations, and will facilitate deeper insight into the plant proteome [99]. (2) VISUAL (vascular cell induction culture system using Arabidopsis leaves) allows the establishment of a transcriptional network affiliated with processes involved in protein interaction, and it unravels the cascades of post-translational modifications by regulating signal propagation and/or transduction within each specific pathway [100]. (3) Adopt a new model system to study vascular biology. We, and a few other groups, have used the *Plantago* family to dissect vasculature-specific transcriptomic alterations due to the ease of vasculature collection from plants in this family [101,102,103]. The analysis of the proteomic changes in the vascular tissue from the *Plantago* family in response to biotic and abiotic stress is not only feasible, it will also bring new insights into the involvement of long-distance signaling between shoot and root in adapting plants to adverse environments.

It is obvious that future efforts should be made to characterize the functions of the identified vasculature proteins. However, caution should be taken because some of the ‘vasculature’ proteins may not be from the vascular tissues, due to the contamination issue. Therefore, it is important to pursue the localization of the studied proteins before an in-depth functional characterization is performed. In recent years, a handful of studies have shown that mRNAs can move to distant tissues via the phloem in order to exert physiological functions. Mobile mRNA in the phloem was reported to contain two catalogues: the species-specific and the universal, conserved across the species [9]. Our preliminary analysis of the literature showed that vascular proteins may also include these two categories. The selection of the proteins for functional analysis should be dependent on the research interest, because proteins involved in a more conserved physiology or species-specific processes may differ. Furthermore, some vascular proteins might be locally synthesized in the vasculature tissue, while others might be transported from the surrounding cells to the vasculature. The possible mechanism related to the transport and regulation remain largely unknown. In addition, the relationship between the vasculature-mobile proteins and mRNAs needs to be elucidated, because some shoot-to-root proteins may directly derive from the shoot as proteins, whereas others may be partially or completely the consequence of the translation of these vasculature-mobile mRNAs.

## Data Availability

Not applicable.
